# Rare mutations in *SQSTM1* modify susceptibility to frontotemporal lobar degeneration

**DOI:** 10.1007/s00401-014-1298-7

**Published:** 2014-06-05

**Authors:** Julie van der Zee, Tim Van Langenhove, Gabor G. Kovacs, Lubina Dillen, William Deschamps, Sebastiaan Engelborghs, Radoslav Matěj, Mathieu Vandenbulcke, Anne Sieben, Bart Dermaut, Katrien Smets, Philip Van Damme, Céline Merlin, Annelies Laureys, Marleen Van Den Broeck, Maria Mattheijssens, Karin Peeters, Luisa Benussi, Giuliano Binetti, Roberta Ghidoni, Barbara Borroni, Alessandro Padovani, Silvana Archetti, Pau Pastor, Cristina Razquin, Sara Ortega-Cubero, Isabel Hernández, Mercè Boada, Agustín Ruiz, Alexandre de Mendonça, Gabriel Miltenberger-Miltényi, Frederico Simões do Couto, Sandro Sorbi, Benedetta Nacmias, Silvia Bagnoli, Caroline Graff, Huei-Hsin Chiang, Håkan Thonberg, Robert Perneczky, Janine Diehl-Schmid, Panagiotis Alexopoulos, Giovanni B. Frisoni, Christian Bonvicini, Matthis Synofzik, Walter Maetzler, Jennifer Müller vom Hagen, Ludger Schöls, Tobias B. Haack, Tim M. Strom, Holger Prokisch, Oriol Dols-Icardo, Jordi Clarimón, Alberto Lleó, Isabel Santana, Maria Rosário Almeida, Beatriz Santiago, Michael T. Heneka, Frank Jessen, Alfredo Ramirez, Raquel Sanchez-Valle, Albert Llado, Ellen Gelpi, Stayko Sarafov, Ivailo Tournev, Albena Jordanova, Eva Parobkova, Gian Maria Fabrizi, Silvia Testi, Eric Salmon, Thomas Ströbel, Patrick Santens, Wim Robberecht, Peter De Jonghe, Jean-Jacques Martin, Patrick Cras, Rik Vandenberghe, Peter Paul De Deyn, Marc Cruts, Kristel Sleegers, Christine Van Broeckhoven

**Affiliations:** 1 Department of Molecular Genetics, VIB, Antwerp, Belgium; 2 Institute Born-Bunge, University of Antwerp, Antwerp, Belgium; 3 Department of Neurology, Antwerp University Hospital, Edegem, Belgium; 4 Institute of Neurology, Neurodegenerative Diseases Group, Medical University of Vienna, Vienna, Austria; 5 Department of Neurology and Memory Clinic, Hospital Network Antwerp Middelheim and Hoge Beuken, Antwerp, Belgium; 6 Department of Pathology and Molecular Medicine, Thomayer Hospital, Prague, Czech Republic; 7 Department of Neurology, First Medical Faculty, Center of Clinical Neurosciences, Charles University in Prague, Prague, Czech Republic; 8 Brain and Emotion Laboratory, Department of Psychiatry, University of Leuven, Louvain, Belgium; 9 Old Age Psychiatry, University Hospitals Leuven and Department of Neurosciences, University of Leuven, Louvain, Belgium; 10 Department of Neurology, University Hospital Ghent, Ghent, Belgium; 11 Center for Medical Genetics, University Hospital Ghent, Ghent, Belgium; 12 Inserm U744, Institut Pasteur de Lille, Université de Lille Nord de France, Lille, France; 13 Department of Neurology, University Hospitals Leuven and University of Leuven, Louvain, Belgium; 14 Laboratory for Neurobiology, Vesalius Research Center, VIB, Louvain, Belgium; 15 NeuroBioGen Lab-Memory Clinic, IRCCS Istituto Centro San Giovanni di Dio Fatebenefratelli, Brescia, Italy; 16 Proteomics Unit, IRCCS Istituto Centro San Giovanni di Dio Fatebenefratelli, Brescia, Italy; 17 Neurology Unit, University of Brescia, Brescia, Italy; 18 III Laboratory of Analysis, Brescia Hospital, Brescia, Italy; 19 Neurogenetics Laboratory, Division of Neurosciences, Center for Applied Medical Research, Universidad de Navarra, Pamplona, Spain; 20 Department of Neurology, Clínica Universidad de Navarra, University of Navarra School of Medicine, Pamplona, Spain; 21 Centro de Investigación Biomédica en Red de Enfermedades Neurodegenerativas, Instituto de Salud Carlos III, Madrid, Spain; 22 Memory Clinic of Fundació ACE, Institut Català de Neurociències Aplicades, Barcelona, Spain; 23 Faculty of Medicine and Institute of Molecular Medicine, University of Lisbon, Lisbon, Portugal; 24 Hospital Santa Maria, Lisbon, Portugal; 25 Department of Neurosciences, Psychology, Drug Research and Child Health (NEUROFARBA), University of Florence, Florence, Italy; 26 Karolinska Institutet, Department of Neurobiology, Care Sciences and Society (NVS), KI-Alzheimer Disease Research Center, Stockholm, Sweden; 27 Genetics Unit, Department of Geriatric Medicine, Karolinska University Hospital, Stockholm, Sweden; 28 Neuroepidemiology and Ageing Research Unit, School of Public Health, Faculty of Medicine, The Imperial College of Science, Technology and Medicine, London, W6 8RP UK; 29 West London Cognitive Disorders Treatment and Research Unit, West London Mental Health Trust, London, TW8 8DS UK; 30 Department of Psychiatry and Psychotherapy, Technische Universität München, 81675 Munich, Germany; 31 Hôpitaux Universitaires de Genève et Université de Genève, Geneva, Switzerland; 32 IRCCS Fatebenefratelli, Brescia, Italy; 33 Department of Neurodegeneration, Hertie Institute for Clinical Brain Research and Centre of Neurology, Tübingen, Germany; 34 German Research Center for Neurodegenerative Diseases (DZNE), Tübingen, Germany; 35 Institute of Human Genetics, Technische Universität München, 81675 Munich, Germany; 36 Institute of Human Genetics, Helmholtz Zentrum München, 85764 Neuherberg, Germany; 37 Department of Neurology, IIB Sant Pau, Hospital de la Santa Creu i Sant Pau, Universitat Autònoma de Barcelona, Barcelona, Spain; 38 Center for Networker Biomedical Research in Neurodegenerative Diseases (CIBERNED), Madrid, Spain; 39 Neurology Department, Centro Hospitalar Universitário de Coimbra, Coimbra, Portugal; 40 Faculty of Medicine, University of Coimbra, Coimbra, Portugal; 41 Center for Neuroscience and Cell Biology, University of Coimbra, Coimbra, Portugal; 42 Clinical Neuroscience Unit, Department of Neurology, University of Bonn, Bonn, Germany; 43 German Center for Neurodegenerative Diseases (DZNE), University of Bonn, Bonn, Germany; 44 Department of Psychiatry and Psychotherapy, University of Bonn, Bonn, Germany; 45 Institute of Human Genetics, University of Bonn, Bonn, Germany; 46 Alzheimer’s Disease and Other Cognitive Disorders Unit, Neurology Department, Hospital Clínic, IDIBAPS, Barcelona, Spain; 47 Neurological Tissue Bank of the Biobanc-Hospital Clinic-Institut d’Investigacions Biomediques August Pi i Sunyer (IDIBAPS), Barcelona, Spain; 48 Department of Neurology, Medical University Sofia, Sofia, Bulgaria; 49 Department of Cognitive Science and Psychology, New Bulgarian University, Sofia, Bulgaria; 50 Department of Biochemistry, Molecular Medicine Center, Medical University, Sofia, Sofia, Bulgaria; 51 Department of Neurological and Movement Sciences, University of Verona, Verona, Italy; 52 Cyclotron Research Centre, University of Liege and Memory Clinic, CHU Liege, Liege, Belgium; 53 Laboratory for Cognitive Neurology, Department of Neurology, University of Leuven and University Hospitals Leuven Gasthuisberg, Louvain, Belgium; 54 Department of Neurology and Alzheimer Research Center, University of Groningen and University Medical Center Groningen, Groningen, The Netherlands

**Keywords:** Sequestosome 1, *SQSTM1*, p62, FTLD, ALS, Rare variants

## Abstract

Mutations in the gene coding for Sequestosome 1 (SQSTM1) have been genetically associated with amyotrophic lateral sclerosis (ALS) and Paget disease of bone. In the present study, we analyzed the *SQSTM1* coding sequence for mutations in an extended cohort of 1,808 patients with frontotemporal lobar degeneration (FTLD), ascertained within the European Early-Onset Dementia consortium. As control dataset, we sequenced 1,625 European control individuals and analyzed whole-exome sequence data of 2,274 German individuals (total *n* = 3,899). Association of rare *SQSTM1* mutations was calculated in a meta-analysis of 4,332 FTLD and 10,240 control alleles. We identified 25 coding variants in FTLD patients of which 10 have not been described. Fifteen mutations were absent in the control individuals (carrier frequency <0.00026) whilst the others were rare in both patients and control individuals. When pooling all variants with a minor allele frequency <0.01, an overall frequency of 3.2 % was calculated in patients. Rare variant association analysis between patients and controls showed no difference over the whole protein, but suggested that rare mutations clustering in the UBA domain of *SQSTM1* may influence disease susceptibility by doubling the risk for FTLD (RR = 2.18 [95 % CI 1.24–3.85]; corrected *p* value = 0.042). Detailed histopathology demonstrated that mutations in *SQSTM1* associate with widespread neuronal and glial phospho-TDP-43 pathology. With this study, we provide further evidence for a putative role of rare mutations in *SQSTM1* in the genetic etiology of FTLD and showed that, comparable to other FTLD/ALS genes, *SQSTM1* mutations are associated with TDP-43 pathology.

**Electronic supplementary material:**

The online version of this article (doi:10.1007/s00401-014-1298-7) contains supplementary material, which is available to authorized users.

## Introduction

Frontotemporal lobar degeneration (FTLD) represents a heterogeneous group of progressive neurodegenerative dementias, caused by local atrophy of frontal and/or temporal lobes. It is one of the most common forms of early-onset dementia (EOD), with the majority of FTLD patients developing disease between 45 and 65 years. About 15 % of FTLD patients present with a motor neuron disease (MND) syndrome, most commonly amyotrophic lateral sclerosis (ALS). Like FTLD, ALS is a neurodegenerative disorder in which loss of motor neurons leads to progressive weakness of the voluntary muscles. FTLD and ALS show important genetic overlap with mutations identified in the same genes, e.g., the common G_4_C_2_ repeat expansion in the chromosome 9 open reading frame 72 gene (*C9orf72*) and less frequently, mutations in the valosin containing protein (*VCP),* fused in sarcoma (*FUS)*, TAR DNA-binding protein (*TARDBP)* and ubiquilin 2 (*UBQLN2*) genes [5, 8, 30, 31, 37].

Sequencing of the gene coding for *sequestosome 1* (*SQSTM1*) in ALS patients identified several rare mutations [7]. Some of these mutations had been associated with Paget disease of bone (PDB [9, 14, 16]), a localized chronic bone disorder characterized by abnormalities of bone architecture and marrow fibrosis, resulting in an osteodystrophia deformans. Of interest, FTLD, PDB and inclusion body myopathy (IBM) had previously been genetically linked by mutations in *VCP* [13, 36, 38]. Recently, rare mutations in *SQSTM1* were also reported in FTLD patients [17, 28]. The observation of rare mutations (1–3 %) in both FTLD and ALS patients suggested an involvement of the protein SQSTM1, also known as p62, in these pathologies possibly through a common disease pathomechanism. The p62 protein is a stress-responsive ubiquitin-binding protein shown to have a role in degradation of polyubiquitinated proteins via the proteasome pathway or autophagic processes [26]. It is present in neuronal and glial ubiquitin-positive inclusions in different tauopathies and synucleinopathies, including Alzheimer disease, FTLD, dementia with Lewy bodies, Parkinson disease, Huntington disease and multiple system atrophy [15, 20, 23]. Also in FTLD, with or without ALS, p62 co-localizes with TDP-43 and FUS in brain and/or spinal cord [2, 6, 32]. Recently, p62 positive but TDP-43 negative immunoreactivity, extending to the pyramidal cell layer of the hippocampus, basal ganglia and cerebellum, has been recognized as a distinctive feature of *C9orf72*-associated FTLD and ALS [1, 34]. Aggregating dipeptide repeats (DPRs), translated from the expanded GGGGCC repeat, were identified as the main component of these inclusions [21, 22]. In the present study, we aimed at determining the genetic contribution of mutations in *SQSTM1* to the etiology of FTLD. Hereto, we analyzed a large study population of 1,808 FTLD patients and compared mutation data to a set of 3,899 European control individuals, as well as 395 European ALS patients.

## Materials and methods

The patient and control cohorts under investigation were ascertained through the European Early-Onset Dementia (EU EOD) consortium (Supplementary table 1) [35]. For the present study, DNA and medical/demographic information on 1,808 FTLD patients, originating from Belgium, Italy, Germany, Spain, Portugal, Sweden, Czech Republic, Bulgaria and Austria, was contributed by members of the consortium. From this patient cohort, 1,706 patients were clinically diagnosed with FTLD and 102 with concomitant FTLD and ALS (FTLD-ALS) (for the remainder of the manuscript, the FTLD group will refer to the 1,706 FTLD patients plus the 102 FTLD-ALS patients). The research question of this study was whether genetic variations in *SQSTM1* affect FTLD risk. Yet, because of the close relationship with FTLD, the contributed patient cohorts also included a number of ALS patients (*n* = 395). Patients were evaluated and diagnosed with FTLD according to the Lund and Manchester group criteria [25] and for ALS according to the revised El Escorial criteria [3]. Clinical diagnoses of behavioral variant frontotemporal dementia (bvFTD) were based on the international consensus criteria by Rascovsky et al. [27] and of progressive supranuclear palsy (PSP) on the National Institute of Neurological Disorders and the Society for PSP criteria [18]. Neuropathological examination was performed in 105 autopsied patients, including 67 with FTLD-TDP, 2 FTLD-UPS, 3 FTLD-tau, 1 FTLD-ni, 4 FTLD unspecified, 21 ALS-TDP and 7 ALS unspecified. Genetic mutation profiling of FTLD- and ALS-associated genes was performed for *C9orf72* (*n* = 2,055), GRN (*n* = 1,024), MAPT (*n* = 854), VCP (*n* = 159), CHMP2B (*n* = 153), TARDBP (*n* = 272) and FUS (*n* = 184) and revealed 150 C9orf72 repeat expansion mutations, 24 GRN, 5 MAPT, 2 VCP, 1 CHMP2B, and 2 FUS mutations. A positive family history was defined for index patients with first- or second-degree relatives with symptoms of dementia or MND. Patients were classified as sporadic when no other affected family members were reported. Patients from whom no information on family history could be obtained were classified as ‘family history undocumented’. As control group, we sequenced 1,625 age- and origin-matched Western-European individuals with no personal or family history of neurodegenerative or psychiatric diseases and a Mini Mental State Examination (MMSE) score >26. We further analyzed whole-exome sequencing (WES) data of 2,274 German non-demented individuals [10, 40]. Together, 3,899 control persons were investigated for coding variants in *SQSTM1.*

For all participants, informed consent for participation in the genetic studies was obtained according to sampling protocols that were approved by the Ethics Committee of the respective hospitals. The protocols for the genetic studies were approved by the Ethics Committee of the University of Antwerp, Belgium.

### Sample quality control

30 µl at 20 ng/µl of genomic DNA (gDNA) was requested, and concentration and purity were checked spectrophotometrically using the Trinean DropSense96 UV/VIS droplet reader for all consortium samples. Gender and DNA fingerprint were determined for all DNA samples using an in-house developed multiplex polymerase-chain reaction (PCR) genomic DNA Fingerprint panel comprising 13 short tandem repeat (STR) markers distributed over multiple autosomal loci—D20S480, D22S1174, D3S1287, D3S1744, D3S1764, D7S672, D7S2426, D8S1746, D14S1005, D20S866, D10S1237, D20S912, D6S965—and two sex chromosome markers—DXS1187, chrom Y: 2655362–2655672—to enable fast and accurate sample identification and gender determination in a single PCR. After selective amplification of 20 ng gDNA under empirically defined reaction conditions, amplification products were size separated on an ABI 3730 automatic sequencer (Applied Biosystems) using GeneScan-600 LIZ (Applied Biosystems) as internal size standard and genotypes were assigned using in-house developed TracI genotyping software (http://www.vibgeneticservicefacility.be). Duplicate samples, gender mismatches and failed samples due to low DNA quality or contamination were excluded, resulting in the final study population of 1,808 FTLD, 395 ALS, and 1,625 control individuals.

### *SQSTM1* sequencing

For the 1,808 FTLD, 395 ALS and 1,625 control individuals, the 8 coding exons and intron–exon boundaries were amplified by PCR of gDNA, followed by Sanger sequencing (NM_003900.4, primers available on request). Sequences were analyzed using the software package NovoSNP [39] and confirmed by visual inspection of the DNA sequence traces. Available WES data on an additional 2,274 German controls were checked for *SQSTM1* coding variants [10, 40].

Genetic variations were further verified in the Database of Single-Nucleotide Polymorphisms (dbSNP Build ID 137; URL http://www.ncbi.nlm.nih.gov/SNP/); the exome variant server (EVS) of the National Heart, Lung, and Blood Institute GO Exome Sequencing Project (Seattle, WA, USA; URL http://evs.gs.washington.edu/EVS/), and the 1,000 Genomes project (URL http://www.1000genomes.org/). The effects of rare coding variations in *SQSTM1* on protein structure and function were predicted using PMUT (http://mmb2.pcb.ub.es:8080/PMut/), SNPs&Go (http://snps.uib.es/snps-and-go//snps-and-go.html) and Provean/SIFT (http://sift.jcvi.org/www/SIFT_enst_submit.html).

### Statistical analysis

**Fig. 1:**
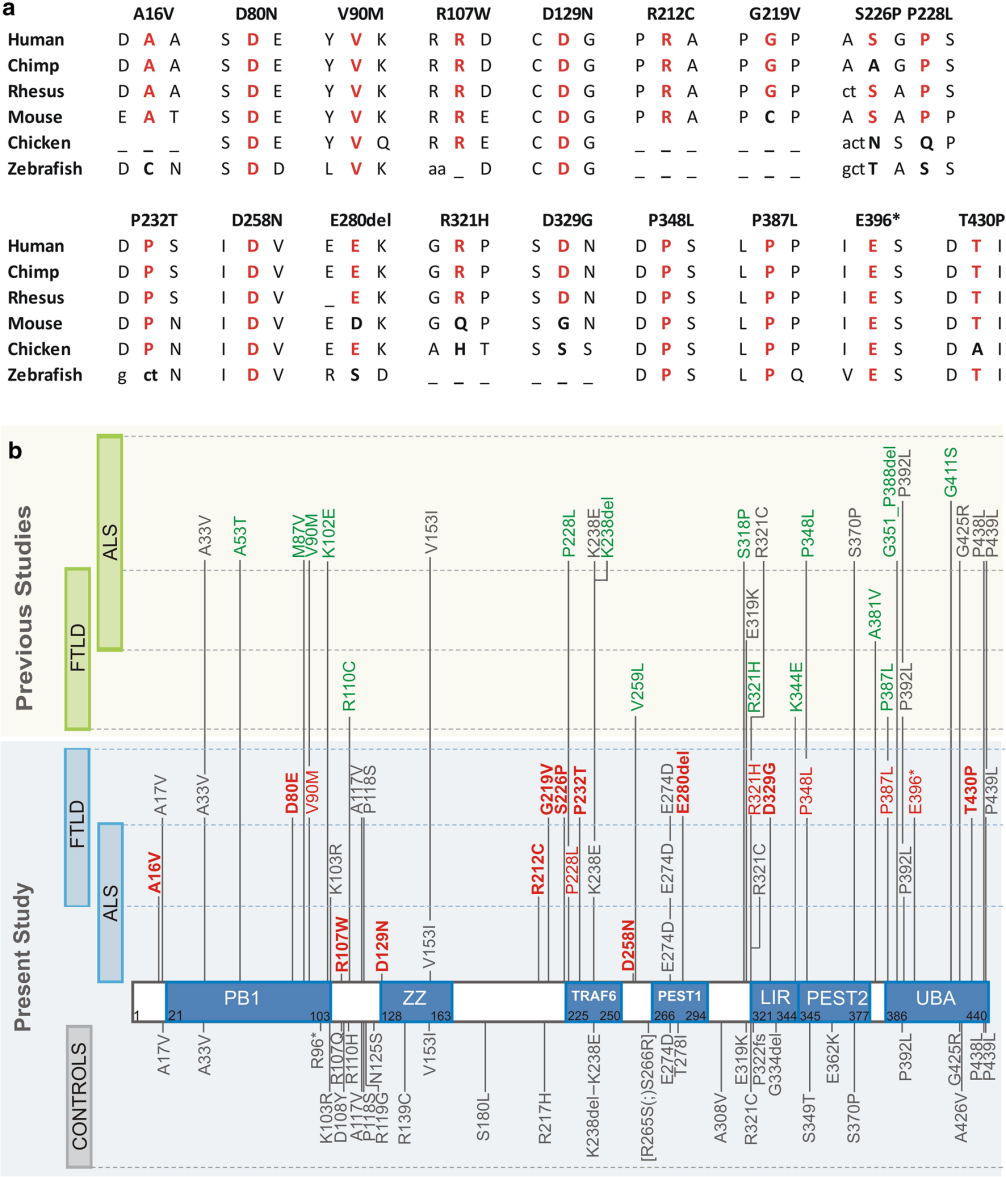
*SQSTM1* mutations identified in FTLD and ALS patient cohorts ascertained with the European EOD consortium. **a** Sequence alignment for patient-specific mutations showing evolutionary conservation across species. **b** In the *blue panel*, *SQSTM1* mutations identified in the present study in patients (*top*) and control individuals (*bottom*) are presented on the primary structure if the p62 protein indicating known functional domains. Mutations absent from tested and published controls are in *red*. Mutations not previously associated with FTLD, ALS, or PDB are in *red* and *bold*. In the *green panel*, *SQSTM1* mutations reported in previous studies are given [7, 11, 17, 28, 29, 33]. Mutations absent from tested and published controls are in *green*. Functional domains according to [11]: PB1 = Phox and Bem1p domain; ZZ = zinc finger motif; TRAF6 = TNF receptor–associated factor 6; LIR = LC3 interaction region; PEST1 = proline (P), glutamic acid (E), serine (S), and threonine (T) domain 1; PEST2 = PEST domain 2; UBA = ubiquitin-associated domain

We determined rare variants as genetic variants with an MAF <0.01 and performed a rare variant burden analysis following a stepwise approach. Alleles of all rare variants were first collapsed across the entire protein. Subsequently, calculations were repeated for rare variants associated with functional domains only. *SQSTM1* functional domains were determined according to [11] (Fig. 1b). Finally, rare variant data were calculated for each of the seven protein domains independently.

Calculations were performed in our source dataset and in a meta-analysis considering all published datasets generated by full exonic sequencing of *SQSTM1* in both FTLD patient and control groups [7, 17, 28] combined with the present study. Overall allele frequencies between patients and control individuals were compared using *χ* ^2^ statistics. In the per domain analyses *p* values were corrected for seven tests, corresponding to the seven functional domains. Rare variant association analysis was limited to the FTLD cohort due to the insufficient power of the ALS cohort to obtain significant association.

### Neuropathology of *SQSTM1*

Formalin fixed, paraffin-embedded tissue blocks from neocortical areas, basal ganglia, thalamus, hippocampus, brainstem and cerebellum were evaluated. In addition to Hematoxylin and Eosin staining, the following monoclonal (mouse) antibodies were used for immunohistochemistry: monoclonal anti-p62 (1:1,000, BD Transduction, Lexington KY, USA), anti-tau AT8 (pS202/pT205, 1:200, Pierce Biotechnology, Rockford, IL, USA), anti-phospho-TDP-43 (pS409/410, 1:2,000, Cosmo Bio, Tokyo, Japan), anti-α-synuclein (1:2,000, clone 5G4, Roboscreen, Leipzig, Germany; specific for disease-associated form), anti-Aβ (1:50, clone 6F/3D, Dako, Glostrup, Denmark). The DAKO EnVision© detection kit, peroxidase/DAB, rabbit/mouse (Dako, Glostrup, Denmark) was used for visualization of the antibody reactions.

## Results

Information on family history was available for 76.2 % (1,378/1,808) of the FTLD patients and 83.0 % (328/395) of ALS patients (Supplementary table 1). In the FTLD group, 35.2 % (636/1,808) had a positive family history while 41.2 % (744/1,808) were considered sporadic patients. In the ALS group, 14.9 % (59/395) had a family history of disease while 68.1 % (269/395) were sporadic patients. Onset age distribution was 63 ± 9.9 years in the FTLD group and 58 ± 13.7 years in the ALS group compared to an age at inclusion of 66 ± 12.4 years in the control group (Supplementary table 1).

### Mutation screen of *SQSTM1* in FTLD

In total we identified 25 rare, heterozygous variations that affected the coding region of *SQSTM1,* including 23 missense mutations, 1 nonsense mutation and one small in-frame deletion (Tables 1, 2, Supplementary table 2; Fig. 1). Fifteen mutations were present in 16 patients and were absent from 3,899 control individuals (Table 1). The majority of the 15 mutations involved highly conserved amino acid residues in SQSTM1 and were predicted to be pathogenic (Fig. 1a, Supplementary table 3). Among the 16 mutation carriers, 7 had a positive family history of disease and 2 patients had concomitant ALS (Table 1). Nine patients carried a novel mutation not previously associated with FTLD, ALS or PDB (p.Ala16Val, p.Asp80Glu, p.Arg212Cys, p.Gly219Val, p.Ser226Pro, p.Pro232Thr, p.Glu280del, p.Asp329Gly and p.Thr430Pro) (Fig. 1b). The remaining six mutations had been reported in ALS (p.Val90Met, p.Pro228Leu and p.Pro348Leu) [7, 28, 29], FTLD (p.Arg321His, p.Pro387Leu) [17] or PDB (p.Pro387Leu, p.(Glu396*)) [28] (Fig. 1b). Except for p.Ala16Val, p.Arg212Cys and p.Gly219Val, all other mutations were located in a predicted functional protein domain of SQSTM1/p62 (Fig. 1b). In addition to the patient-only mutations, we identified another 10 missense mutations (p.Ala17Val, p.Ala33Val, p.Lys103Arg, p.Ala117Val, p.Pro118Ser, p.Lys238Glu, p.Glu274Asp, p.Arg321Cys, p.Pro392Leu, p.Pro439Leu) that were also present in control individuals at low frequency (Table 2). Except for p.Glu274Asp (MAF of 0.024), all were present in less than 1 % of control individuals. p.Ala17Val was a novel variation not previously reported in FTLD, ALS and/or PDB patients. We identified another 21 variants present in control individuals only (Table 3). When considering all rare variants (MAF <0.01) observed in FTLD patients an overall frequency was calculated of 3.2 % (58/1,808).

**Table 1:** *SQSTM1* mutations present only in patients and associated clinical phenotypes

Mutation	Functional domain	Origin	Gender	Clinical diagnosis	Sub-diagnosis^b^	Family history	Age at onset (years)	Age at death (years)
FTLD								
p.Ala16Val^a^		Italian	M	FTLD-ALS		U	71	74
p.Asp80Glu^a^	PB1	Italian	F	FTLD	bvFTD	F	71	85
p.Val90Met	PB1	Portuguese	F	FTLD	bvFTD	U	41	
p.Arg212Cys^a^		Austrian	M	FTLD-ALS		F	63	66
p.Gly219Val^a^		Portuguese	M	FTLD	bvFTD	F	52	
p.Ser226Pro^a^	TRAF6	Spanish	M	FTLD	bvFTD	S	61	
p.Pro228Leu	TRAF6	German	M	FTLD	bvFTD	S	57	
p.Pro232Thr^a^	TRAF6	Portuguese	F	FTLD^c^	bvFTD	F	55	68
p.Glu280del^a^	PEST1	Italian	F	FTLD	PSP	F	73	
p.Arg321His	LIR	Italian	F	FTLD		U	68	
p.Asp329Gly^a^	LIR	Spanish	M	FTLD	bvFTD	U	78	84
p.Pro348Leu	PEST2	Italian	M	FTLD	PNFA	F	74	
p.Pro387Leu	UBA	Italian	F	FTLD	PNFA	F	65	
p.Pro387Leu	UBA	Italian	M	FTLD	bvFTD	S	66	
p.(Glu396*)	UBA	Czech	M	FTLD	bvFTD	S	43	47
p.Thr430Pro^a^	UBA	Portuguese	M	FTLD	bvFTD	S	58	63
ALS								
p.Arg107Trp^a^		Spanish	F	ALS	MND	S	58	62
p.Asp129Asn^a^	ZZ	Flemish	M	ALS		S	62	
p.Asp258Asn^a^		German	F	ALS		F	52	62

Functional domains according to [11] (Fig. 1b)

*bvFTD* behavioral variant frontotemporal dementia, *MND* motor neuron disease, *PSP* progressive supranuclear palsy, *PNFA* progressive non-fluent aphasia, *F* familial, *S* sporadic, *U* family history undocumented

^a^Indicates variants not previously associated with ALS, FTLD or PDB [7, 11, 17, 28, 29, 33]. For a complete description of SQSTM1 mutations, see Supplementary table 2

^b^Clinical subdiagnosis is given where documented

^c^After revision of the medical records of the mutation carriers, a diagnosis of possible PDB was made in hindsight in this patient

**Table 2:** *SQSTM1* mutations present in patients and control individuals

Mutation	Functional domain	FTLD *n* = 1,808	ALS *n* = 395	Controls *n* = 3,899
p.Ala17Val^a^		1		1
p.Ala33Val	PB1	1		1
p.Lys103Arg	PB1	1		2
p.Ala117Val		1		3
p.Pro118Ser		1		2
p.Val153Ile	ZZ		1	3
p.Lys238Glu	TRAF6	17	6	14
p.Glu274Asp	PEST1	109	22	79
p.Arg321Cys	LIR	3	1	1
p.Pro392Leu	UBA	15	3	11
p.Pro439Leu	UBA	2		2

Functional domains according to [11] (Fig. 1b)

^a^Indicates variants not previously associated with ALS, FTLD or PDB [7, 11, 17, 28, 29, 33]. For a complete description of the SQSTM1 mutations, see Supplementary table 2

**Table 3:** Descriptives of the *SQSTM1* variants found in control individuals only

On cDNA level	Exon	On protein level	Functional domain	dbSNP
NM_003900.4:c.286C>T	Exon 2	NP_003891.1:p.(Arg96*)	PB1	
NM_003900.4:c.308A>G	Exon 3	NP_003891.1:p.Arg107Gln		
NM_003900.4:c.322G>T	Exon 3	NP_003891.1:p.Asp108Tyr		
NM_003900.4:c.329G>A	Exon 3	NP_003891.1:p.Arg110His		
NM_003900.4:c.355C>G	Exon 3	NP_003891.1:p.Arg119Gly		
NM_003900.4:c.374A>G	Exon 3	NP_003891.1:p.Asn125Ser		
NM_003900.4:c.415C>T	Exon 3	NP_003891.1:p.Arg139Cys	ZZ	
NM_003900.4:c.539C>T	Exon 4	NP_003891.1:p.Ser180Leu		
NM_003900.4:c.650G>A	Exon 4	NP_003891.1:p.Arg217His		
NM_003900.4:c.711_713delGAA	Exon 5	NP_003891.1:p.lys238del	TRAF6	
NM_003900.4:c.795_796delinsTT	Exon 6	NP_003891.1:p.[Arg265Ser(;) Ser266Arg]	PEST1	
NM_003900.4:c.833C>T	Exon 6	NP_003891.1:p.Thr278Ile	PEST1	rs200445838
NM_003900.4:c.923C>T	Exon 6	NP_003891.1:p.Ala308Val		
NM_003900.4:c.955G>A	Exon 6	NP_003891.1:p.Glu319lys		rs61748794
NM_003900.4:c.965_966delCT	Exon 6	NP_003891.1:p.Pro322 fs	LIR	
NM_003900.4:c.1001_1003delGAG	Exon 7	NP_003891.1:p.Gly334del	LIR	
NM_003900.4:c.1045T>A	Exon 7	NP_003891.1:p.Ser349Thr	PEST2	
NM_003900.4:c.1084G>A	Exon 7	NP_003891.1:p.Glu362Lys		
NM_003900.4:c.1108T>C	Exon 7	NP_003891.1:p.Ser370Pro		rs143956614
NM_003900.4:c.1273G>A	Exon 8	NP_003891.1:p.Gly425Arg	UBA	
NM_003900.4:c.1277C>T	Exon 8	NP_003891.1:p.Ala426Val	UBA	

### Mutation screen of *SQSTM1* in ALS

For comparison with our FTLD findings, we analyzed the 395 ALS patients that were concurrently ascertained within the EU EOD consortium (Fig. 1). We identified three novel missense mutations present only in the ALS patients (p.Arg107Trp, p.Asp129Asn and p.Asp258Asn) (Table 1) and five other missense mutations (p.Val153Ile, p.Lys238Glu, p.Glu274Asp, p.Arg321Cys and p.Pro392Leu) also present in control individuals at low frequency (Table 2, Supplementary table 2).

### Association of rare *SQSTM1* variants

We performed a burden analysis collapsing all rare variants with an MAF <0.01 across the whole protein. The same frequency of rare alleles was calculated for FTLD patients 0.016 (58/3,616 rare variant alleles) as for control individuals 0.016 (125/7,798; *p* value = 0.997, n.s.). We repeated the same analysis for rare variants present in functional domains only, resulting in a shift in allele frequencies of 0.014 (52/3,616) in FTLD versus 0.011 (84/7,798) controls, although not reaching statistical significance (*p* = 0.098). To increase genetic power, we considered all published datasets that were generated by full exonic sequencing of *SQSTM1* in both FTLD patient and control groups [7, 17, 28] and included the data in a meta-analysis with the present study comprising mutant allele frequencies in 4,332 FTLD and 10,240 control alleles. Burden analysis of all rare variants across the protein showed again no increase in patients (70/4,332 = 0.016 in FTLD versus 142/10,240 = 0.014 in controls; *p* value = 0.291, n.s.), but for the rare variants associated with functional domains a marked significant increase could be calculated of 0.014 (61/4,332 rare variant alleles) in FTLD patients versus 0.009 (97/10,240) in control individuals (relative risk overall (RR) = 1.49 [95 % CI 1.08–2.06]; *p* value = 0.014).

Subsequently, we investigated which of the functional domains were most contributing to the association. Rare variant data were calculated for each of the seven protein domains. In our source, FTLD cohort significant association was found with the C-terminal ubiquitin-associated (UBA) domain in patients (21/3,616 = 0.006 rare variant alleles) versus control individuals (20/7,798 = 0.003) (RR = 2.27 [95 % CI 1.23–4.20]; *p* value = 0.007, corrected *p* value = 0.049). In the meta-dataset, statistically significant clustering in the UBA domain was confirmed in FTLD (23/4,332 = 0.005 rare variant alleles) versus control individuals (25/10,240 = 0.002) (RR = 2.18 [95 % CI 1.24–3.85]; *p* value = 0.006, corrected *p* value = 0.042). In addition, also the LC3 interaction region (LIR) domain showed suggestive clustering of rare variants in FTLD (8/4,332 = 0.002) versus control individuals (5/10,240 = 0.0005) (RR = 3.79 [95 % CI 1.24–11.58]; *p* value = 0.012, corrected *p* value = 0.084).

### Histopathology associated with *SQSTM1* mutations

**Fig. 2:**
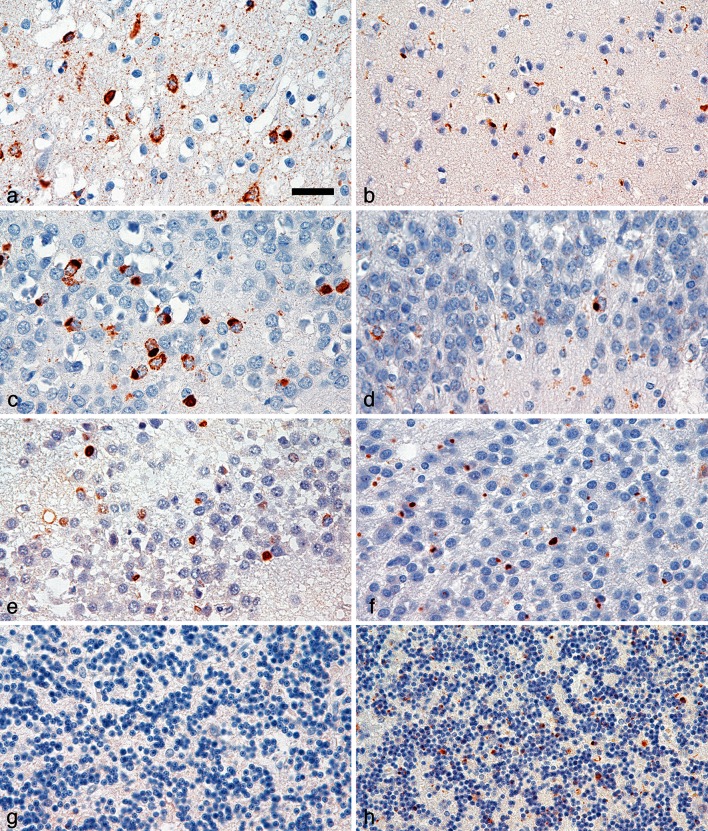
Neuropathology observed in *SQSTM1* mutation carriers. Immunostaining for phospho-TDP-43 in the temporal cortex (**a**, **b**) and in the granule cells of the dentate gyrus (**c**, **d**) in a patient with a *SQSTM1* p.(Glu396*) mutation (**a**, **c**) and a second patient with a *SQSTM1* p.Arg212Cys—*C9orf72* double mutation (**b**, **d**). Notably, p62 immunoreactivity is less in the dentate gyrus (**e**, **f**) and lacking in the cerebellar granule cell layer (**g**, **h**) in the p.(Glu396*) case (**e**, **g**) as compared to the p.Arg212Cys patient with the additional *C9orf72* repeat expansion mutation (**f**, **h**). Scale bar represents 25 µm for all

Detailed histopathology was performed on autopsy brain of two carriers of a nonsense mutation p.(Glu396*) located in the UBA domain and a missense mutation p.Arg212Cys in a patient who also carried a *C9orf72* repeat expansion. Both cases showed widespread phospho-TDP-43 immunoreactive inclusions in neurons and glial cells. In the p.(Glu396*) carrier, frontal and temporal cortical areas mainly displayed neuronal cytoplasmic inclusions. Neuronal cytoplasmic inclusions were further abundant in the granule cells of the dentate gyrus. In addition, many glial phospho-TDP-43 inclusions were seen in the white matter. Although this brain suffered severe ischemic/hypoxic damage, inclusion bodies were clearly recognized as a distinctive feature. In contrast, neuropathological features of the p.Arg212Cys carrier revealed alterations reminiscent of the *C9orf72* pathology, including the characteristic p62-positive but phospho-TDP-43-negative inclusions in the hippocampal pyramidal layer and cerebellar granule cells (Fig. 2).

## Discussion

Previous studies in ALS (total *n* = 895) [7, 11, 28, 33], and FTLD (total *n* = 358) [17, 28] estimated the frequency of *SQSTM1* coding mutations at 2.42–3.28 % in ALS and 1.76–2.13 % in FTLD. Because of this relatively low prevalence, large patient and control groups are needed to obtain a reliable estimation of the mutation frequency of *SQSTM1*. Mutations in *SQSTM1* were first described in patients with PDB [16]. Up to one-third of patients with familial PDB are explained by a *SQSTM1* mutation, with p.Pro392Leu as the most commonly observed mutation [4]. A relationship between PDB and FTLD has been previously demonstrated by the identification of causal mutations in *VCP* in families segregating the rare syndrome of inclusion body myopathy with Paget disease of bone and frontotemporal lobar degeneration (IBMPFD) [38]. Later studies demonstrated that *VCP* mutations express significant clinical heterogeneity and patients can present with all, a combination of two or just one of the three core phenotypes of IBMPFD [24, 36]. Also more recently, WES identified a *VCP* mutation segregating in a family with ALS and subsequent screenings in ALS patients identified additional *VCP* mutations [12].

In this study, we analyzed a European cohort of 1,808 FTLD patients for mutations in *SQSTM1* and identified 25 mutations in the coding sequence of which 15 mutations in 16 patients were absent from 3,899 control persons. This resulted in a mutation frequency for *SQSTM1* of 0.9 % overall and 1.1 % in familial FTLD patients. Some of the mutations are listed in dbSNP Short Genetic Variations database, the 1,000 Genomes Project database or the exome variant server (EVS), yet at very low frequencies of MAF ≤0.001 (Supplementary table 2). The *SQSTM1* mutation frequency that we calculated in FTLD is lower than in previous studies. This can potentially be explained by the fact that we excluded some of the previously reported patient-only mutations from our calculations since they were present in our extended control group, though at low frequencies <0.01 % (Fig. 1b). This was the case for p.Arg321Cys, p.Ser370Pro, p.Gly425Arg [7]; p.Ala33Val, p.Pro392Leu [7, 17]; p.Lys238Glu, p.Glu319Lys [28]; p.Pro439Leu [11]; and p.Val153Ile [7, 29]. In Table 4, all *SQSTM1* mutations are listed that have been published [7, 11, 17, 28, 29, 33] and were absent from published and tested control persons. When we considered all rare variants (MAF < 0.01), indifferent of whether or not they appeared in control individuals, we obtained a mutation frequency of 3.2 % in FTLD patients which is the same as calculated in the pooled data analysis of the present and published FTLD cohorts [17, 28]. Overall, no statistical association in patients versus control individuals was observed when pooling all rare *SQSTM1* variants, not in our study nor in the meta-analysis with all published patient and control datasets [7, 17, 28]. Yet, when considering only domain-associated variants, a trend toward association was observed in our study and a significant increase in patients was reached in the more powerful meta-analysis dataset. Per domain analysis indicated that association was driven by the UBA domain and possibly also the LIR domain. The C-terminal UBA domain, which is primarily affected in PDB, contained significant more variants in FTLD patients when compared to control individuals in both our study and the meta-analysis (0.5 versus 0.2 %) (RR_meta_ = 2.18 [95 % CI 1.24–3.85]; nominal *p* value_meta_ = 0.006; corrected *p* value_meta_ = 0.042). In the LIR domain through which SQSTM1 binds the autophagy effector protein LC3, 0.2 % of FTLD patients carried a mutation versus 0.05 % of control individuals (RR_meta_ = 3.79 [95 % CI 1.24–11.58]; nominal *p* value_meta_ = 0.012; corrected *p* value_meta_ = 0.084). In contrast to Rubino and colleagues who sequenced up to 1,700 bp into the *SQSTM1* promoter and detected 4 variants in 4 out of 170 FTLD patients absent from 145 control individuals (c. −1,221 G>A, c. −1,165 C>T, c. −1,153 C>G, c. −673 T>C) [28], we did not investigate the *SQSTM1* promoter for rare variants.

**Table 4:** *SQSTM1* mutations published in previous studies

Mutation	Functional domain	FTLD	ALS	Origin	Study
p.Ala53Thr	PB1		1	Japanese	Hirano et al. [11]
p.Met87Val	PB1		1	French	Teyssou et al. [33]
p.Val90Met^a^	PB1		1	Japanese	Shimizu et al. [29]
p.Lys102Glu	PB1		1	French	Teyssou et al. [33]
p.Arg110Cys		2	1	French	Le Ber et al. [17]
p.Pro228Leu	TRAF6		1	Euro-American	Fecto et al. [7]
p.Lys238del	TRAF6		1	Euro-American	Fecto et al. [7]
p.Val259Leu		1		Italian	Rubino et al. [28]
p.Ser318Pro			1	Euro-American	Fecto et al. [7]
p.Arg321His	LIR	2	1	French	Le Ber et al. [17]
p.Lys344Glu	LIR	1		Italian	Rubino et al. [28]
p.Pro348Leu	PEST2		1	Italian	Rubino et al. [28]
p.Ala381Val		1	1	French	Le Ber et al. [17]
p.Pro387Leu	UBA	1		French	Le Ber et al. [17]
p.G351_P388del	UBA		1	French	Teyssou et al. [33]
p.Gly411Ser	UBA		1	Euro-American	Fecto et al. [7]

Mutations published in previous studies [7, 11, 17, 28, 29, 33] absent from published control persons or control persons tested in the present study are listed

^a^This mutation was compound heterozygous with p.Val153Ile in one Japanese ALS patient [29], p.Val153Ile was also observed in 3 control individuals of the present study. Fecto et al. [7] tested 546 ALS and 724 controls. Rubino et al. [28] tested 170 FTLD, 124 ALS, and 145 controls. Teyssou et al. [33] tested 164 ALS and 360 controls. Hirano et al. [11] tested 61 ALS and 500 controls. Le Ber et al. [17] tested 188 FTLD, 164 ALS, and 352 controls. Shimizu et al. [29] tested 1 ALS and 189 controls

The pathogenicity of *SQSTM1* mutations, even those that were absent from control individuals, remains unclear at this stage. Most of the patient-only mutations involved highly conserved amino acid residues, with 75 % of the mutations residing in predicted functional domains. In silico prediction programs of amino acid changes indicated that all mutations might be pathogenic by one or more of the in silico programs. Also, the three mutations that resided outside predicted domains were scored as deleterious by one to all four in silico programs. Of course, these in silico predictions are merely indicative and should be interpreted with caution. In contrast to PDB-associated mutations which cluster at the UBA domain [11, 16], FTLD mutations are mostly distributed throughout the protein as also previously reported for ALS, though significantly more mutations seem to be located in the UBA domain. Further, Le Ber and colleagues provided evidence for co-segregation with FTLD for two mutations in the UBA domain, p.Pro387Leu and p.Pro392Leu [17]. The p.Pro387Leu mutation was also present in two unrelated Italian FTLD patients in our study who did not share a common haplotype, suggesting a recurrent de novo mutation. No DNA of relatives of these two mutation carriers was available to test for co-segregation. Three mutation carriers (p.Ala16Val, p.Arg212Cys, p.Gly219Val) also carried a pathogenic *C9orf72* repeat expansion. At this stage, we have no clear indication that the co-existence of these mutations influences clinical expression of disease. Onset ages were not different from those in patients carrying only a *SQSTM1* mutation. In a Japanese sporadic late-onset ALS patient with pathology confirmed predominant lower motor neuron disease, a compound heterozygous *SQSTM1* mutation was detected, p.[Val90Met(;)Val153Ile] [29]. We did not observe compound heterozygous *SQSTM1* mutation carriers, though both mutations present in the Japanese patient were also observed in our study, p.Val90Met in one FTLD patient and p.Val153Ile in one other FTLD patient and three control individuals. One could hypothesize that the rare *SQSTM1* variants act as high to intermediate penetrant risk alleles and in case of the Japanese patient both had to be present to exert the clinical profile of ALS.

The hallmark lesions of FTLD-TDP are neuronal and glial inclusions with positive immunoreactivity for phospho-TDP-43. In addition to sporadic patients, until now mutations in *VCP*, *GRN* and *C9orf72* have been associated consistently with TDP-43 pathology, while TARDBP mutations are less frequently observed in FTLD [19]. Here, we provide histopathological evidence that *SQSTM1* mutations are associated with TDP-43 pathological inclusions. Interestingly, the patient with only a *SQSTM1* mutation (p.Glu396*) showed widespread neuronal and glial phospho-TDP-43 pathology. The patient (p.Arg212Cys) who carried also a *C9orf72* mutation showed *C9orf72*-related pathology without other noticeable distinctive features. Potential explanations could be that the *C9orf72* mutation masked the effect of the *SQSTM1*mutation, or that the *C9orf72* pathology dominates the *SQSTM1* pathology. Also the *SQSTM1* p.Arg212Cys mutation may well have a lower penetrance and be associated with more subtle pathological changes than the (p.Glu396*) mutation located in the UBA domain. These questions merit further investigation and more *SQSTM1*-positive pathologies will need to be evaluated to resolve these issues. Since mutations in *SQSTM1* were first linked to PDB, we carefully reexamined medical records of all carriers of patient-only *SQSTM1* mutations for indications of (sub) clinical symptoms of PDB. Only in one Portuguese patient carrying the p.Pro232Thr mutation located in the TRAF6 domain, the diagnosis of possible PDB could be made at hindsight. We, however, cannot exclude that the presence of PDB might have been missed in the other patients since PDB often remains asymptomatic and a diagnosis requires radiography. Likewise we cannot exclude that some of the control individuals, who were selected based on absence of dementia-related symptoms, may have been at risk for/or suffered from PDB, in particular, those with the PDB-associated *SQSTM1* p.P392L mutation.

In conclusion, our study represents one of the largest screening efforts of FTLD patients for mutations in *SQSTM1*. Further, we combined the data from our screening with that of published datasets in a meta-analysis of rare *SQSTM1* variants in a total of 4,432 FTLD and 10,240 control alleles. Both our study and the meta-analysis calculated a mutation frequency of 3.2 % in FTLD patients. Also, the meta-analysis suggested that rare mutations clustering in the UBA domain of *SQSTM1* may influence disease susceptibility by doubling the risk for FTLD. Histopathology of autopsied brain of *SQSTM1* mutation carriers demonstrated a widespread neuronal and glial phospho-TDP-43 pathology. Taken together, our findings provide additional evidence that *SQSTM1* is implicated in the pathogenicity of FTLD/ALS spectrum diseases.

## Electronic supplementary material

Below is the link to the electronic supplementary material.

Supplementary material 1 (DOCX 41 kb)

## Appendix

*BELNEU consortium* Jonathan Baets (Department of Molecular Genetics, VIB, Antwerp, Belgium; Institute Born-Bunge, University of Antwerp, Antwerp, Belgium; Antwerp University Hospital, Edegem, Belgium); Dirk Nuytten (Hospital Network Antwerp Stuivenberg, Antwerp, Belgium); Jan De Bleecker (University Hospital Ghent, Ghent, Belgium); Jan Versijpt, Alex Michotte (University Hospital Brussels, Brussels, Belgium); Adrian Ivanoiu (Saint-Luc University Hospital, Brussels, Belgium); Olivier Deryck, Bruno Bergmans (AZ Sint-Jan Brugge, Bruges, Belgium); Christiana Willems (Jessa Hospital, Hasselt, Belgium).

*EU EOD consortium* Anna Paterlini (Proteomics Unit, IRCCS Istituto Centro San Giovanni di Dio Fatebenefratelli, Brescia, Italy); Elena Alonso (Neurogenetics Laboratory, Division of Neurosciences, Center for Applied Medical Research, Universidad de Navarra, Pamplona, Spain); Manuel Seijo-Martínez (Department of Neurology, Hospital do Salnés, Pontevedra, Spain); Ramon Rene, Jordi Gascon, Jaume Campdelacreu (Department of Neurology, Hospital de Bellvitge, Barcelona, Spain); Madalena Martins, Mafalda Matos, André Janeiro, Ana Verdelho (Faculty of Medicine and Institute of Molecular Medicine, University of Lisbon, Portugal); Irene Piaceri (Department of Neurosciences, Psychology, Drug Research and Child Health (NEUROFARBA) University of Florence, Florence, Italy); Jenny Björkström, Anne Kinhult Ståhlbom, Marie Fallström (Department of Geriatric Medicine, Genetics unit, Karolinska University Hospital, Stockholm, Sweden); Charlotte Forsell, Anna-Karin Lindström, Lena Lilius (Karolinska Institutet, Department of Neurobiology, Care Sciences and Society (NVS), KI-Alzheimer Disease Research Center, Stockholm, Sweden); Inger Nennesmo (Department of Clinical Pathology and Cytology, Karolinska University Hospital, Stockholm, Sweden); Laura Fratiglioni (Aging Research Center, Department of Neurobiology, Care Sciences and Society (NVS), Karolinska Institutet and Stockholm University, Stockholm, Sweden);Tamara Eisele (Department of Psychiatry and Psychotherapy, Technische Universität München, 81675 München, Germany); Thomas Wieland (Institute of Human Genetics, Technische Universität München, 81675 Munich, Germany); Ricard Rojas-García, Marc Suárez-Calvet (Department of Neurology, IIB Sant Pau, Hospital de la Santa Creu i Sant Pau, Universitat Autònoma de Barcelona, Barcelona, Spain; Center for Networker Biomedical Research in Neurodegenerative Diseases (CIBERNED), Madrid, Spain); João Massano (Department of Neurology Centro Hospitalar São João, Portugal; Department of Clinical Neuroscience and Mental Health, Faculty of Medicine University of Porto, Porto, Portugal); Maria Helena Ribeiro (Faculty of Medicine, University of Coimbra, Coimbra, Portugal); Catarina Oliveira (Center for Neuroscience and Cell Biology, University of Coimbra, Coimbra, Portugal); Jose L Molinuevo, Anna Antonell (Alzheimer’s disease and other cogntive disorders unit. Neurology department, Hospital); Robert Rusina, Zdenek Rohan (Department of Pathology and Molecular Medicine, Thomayer Hospital, Prague, Czech Republic); Robert Rusina (Center of Clinical Neurosciences, Department of Neurology, First Medical Faculty, Charles University in Prague, Czech Republic); Tiziana Cavallaro (Department of Neuroscience, AOUI Verona, Verona, Italy).
