# Supplementary material for: Rare mutations in SQSTM1 modify susceptibility to frontotemporal lobar degeneration
Source: Acta Neuropathol. 2014 Jun 5;128(3):397–410. doi: 10.1007/s00401-014-1298-7 (PMC4131163; doi:10.1007/s00401-014-1298-7)
Supplement: Supplementary file 1 — Supplementary material 1 (DOCX 41 kb) [file 401_2014_1298_MOESM1_ESM.docx]

**Rare mutations in *SQSTM1* modify susceptibility to frontotemporal lobar degeneration**

Julie van der Zee 1,2; Tim Van Langenhove 1,2,3; Gabor G. Kovacs 4; Lubina Dillen 1,2; William Deschamps 1,2; Sebastiaan Engelborghs 2,5; Radoslav Matej 6,7; Mathieu Vandenbulcke 8,9; Anne Sieben 1,2,10; Bart Dermaut 10, 11,12; Katrien Smets 1,2,3; Philip Van Damme 13,14; Céline Merlin 1,2; Annelies Laureys 1,2; Marleen Van Den Broeck 1,2; Maria Mattheijssens 1,2; Karin Peeters 1,2; Luisa Benussi 15; Giuliano Binetti 15; Roberta Ghidoni 16; Barbara Borroni 17; Alessandro Padovani 17; Silvana Archetti 18; Pau Pastor 19,20,21; Cristina Razquin 19; Sara Ortega-Cubero 19,20,21; Isabel Hernández 22; Mercè Boada 22; Agustín Ruiz 22; Alexandre de Mendonça 23; Gabriel Miltenberger-Miltényi 23; Frederico Simões do Couto 23,24; Sandro Sorbi 25; Benedetta Nacmias 25; Silvia Bagnoli 25; Caroline Graff 26,27; Huei-Hsin Chiang 26,27; Håkan Thonberg 27; Robert Perneczky 28,29,30; Janine Diehl-Schmid 30; Panagiotis Alexopoulos 30; Giovanni B Frisoni 31,32; Christian Bonvicini 32; Matthis Synofzik 33,34; Walter Maetzler 33,34; Jennifer Müller vom Hagen 33,34; Ludger Schöls 33,34; Tobias B. Haack 35,36; Tim M. Strom 35,36; Holger Prokisch 35,36; Oriol Dols-Icardo 37,38; Jordi Clarimón 37,38; Alberto Lleó 37,38; Isabel Santana 39,40; Maria Rosário Almeida 41; Beatriz Santiago 39; Michael T. Heneka 42,43; Frank Jessen 43,44; Alfredo Ramirez 44,45; Raquel Sanchez-Valle 46; Albert Llado 46; Ellen Gelpi 47; Stayko Sarafov 48; Ivailo Tournev 48,49; Albena Jordanova 1,2,50; Eva Parobkova 6; Gian Maria Fabrizi 51; Silvia Testi 51; Eric Salmon 52; Thomas Ströbel 4; Patrick Santens 10; Wim Robberecht 13,14; Peter De Jonghe 1,2,3; Jean-Jacques Martin 1,2; Patrick Cras 2,3; Rik Vandenberghe 9,53; Peter Paul De Deyn 2,5,54; Marc Cruts 1,2; Kristel Sleegers 1,2; Christine Van Broeckhoven 1,2,^✝^; on behalf of the BELNEU consortium* and of the EU EOD consortium**

1 Department of Molecular Genetics, VIB, Antwerp, Belgium

2 Institute Born-Bunge, University of Antwerp, Antwerp, Belgium

3 Department of Neurology, Antwerp University Hospital, Edegem, Belgium

4 Institute of Neurology, Neurodegenerative Diseases group, Medical University of Vienna, Vienna, Austria

5 Department of Neurology and Memory Clinic, Hospital Network Antwerp Middelheim and Hoge Beuken, Antwerp, Belgium

6 Department of Pathology and Molecular Medicine, Thomayer Hospital, Prague, Czech Republic

7 Center of Clinical Neurosciences, Department of Neurology, First Medical Faculty, Charles University in Prague, Czech Republic

8 Brain and Emotion Laboratory, Department of Psychiatry, University of Leuven, Leuven, Belgium

9 Old Age Psychiatry, University Hospitals Leuven and Department of Neurosciences, University of Leuven, Belgium

10 Department of Neurology, University Hospital Ghent, Ghent, Belgium

11 Center for Medical Genetics, University Hospital Ghent, Ghent, Belgium

12 Inserm U744, Institut Pasteur de Lille, Université de Lille Nord de France, 59019 Lille, France

13 Department of Neurology, University Hospitals Leuven and University of Leuven, Leuven, Belgium

14 Laboratory for Neurobiology, Vesalius Research Center, VIB, Leuven, Belgium

15 NeuroBioGen Lab - Memory Clinic, IRCCS Istituto Centro San Giovanni di Dio Fatebenefratelli, Brescia, Italy

16 Proteomics Unit, IRCCS Istituto Centro San Giovanni di Dio Fatebenefratelli, Brescia, Italy

17 Neurology Unit, University of Brescia, Brescia, Italy

18 III Laboratory of Analysis, Brescia Hospital, Brescia, Italy

19 Neurogenetics Laboratory, Division of Neurosciences, Center for Applied Medical Research, Universidad de Navarra, Pamplona, Spain

20 Department of Neurology, Clínica Universidad de Navarra, University of Navarra School of Medicine, Pamplona, Spain

21 Centro de Investigación Biomédica en Red de Enfermedades Neurodegenerativas, Instituto de Salud Carlos III, Madrid, Spain

22 Memory Clinic of Fundació ACE, Institut Català de Neurociències Aplicades, Barcelona, Spain

23 Faculty of Medicine and Institute of Molecular Medicine, University of Lisbon, Lisbon, Portugal

24 Hospital Santa Maria, Lisbon, Portugal

25 Department of Neurosciences, Psychology, Drug Research and Child Health (NEUROFARBA) University of Florence, Florence, Italy

26 Karolinska Institutet, Department of Neurobiology, Care sciences and society (NVS), KI-Alzheimer Disease Research Center, Stockholm, Sweden

27 Department of Geriatric Medicine, Genetics unit, Karolinska University Hospital, Stockholm, Sweden

28 Neuroepidemiology and Ageing Research Unit, School of Public Health, Faculty of Medicine, The Imperial College of Science, Technology and Medicine, London, W6 8RP, UK

29 West London Cognitive Disorders Treatment and Research Unit, West London Mental Health Trust, London, TW8 8DS, UK

30 Department of Psychiatry and Psychotherapy, Technische Universität München, 81675 München, Germany

31 Hôpitaux Universitaires de Genève et Université de Genève, Genève, Switzerland

32 IRCCS Fatebenefratelli, Brescia, Italy

33 Department of Neurodegeneration, Hertie Institute for Clinical Brain Research and Centre of Neurology, Tübingen, Germany

34 German Research Center for Neurodegenerative Diseases (DZNE), Tübingen, Germany

35 Institute of Human Genetics, Technische Universität München, 81675 Munich, Germany

36 Institute of Human Genetics, Helmholtz Zentrum München, 85764 Neuherberg, Germany

37 Department of Neurology, IIB Sant Pau, Hospital de la Santa Creu i Sant Pau, Universitat Autònoma de Barcelona, Barcelona, Spain

38 Center for Networker Biomedical Research in Neurodegenerative Diseases (CIBERNED), Madrid, Spain

39 Neurology Department, Centro Hospitalar Universitário de Coimbra, Coimbra, Portugal

40 Faculty of Medicine, University of Coimbra, Coimbra, Portugal

41 Center for Neuroscience and Cell Biology, University of Coimbra, Coimbra, Portugal

42 Clinical Neuroscience Unit, Department of Neurology, University of Bonn, Germany

43 German Center for Neurodegenerative Diseases (DZNE), University of Bonn, Germany

44 Department of Psychiatry and Psychotherapy, University of Bonn, Bonn, Germany

45 Institute of Human genetics, University of Bonn, Bonn, Germany

46 Alzheimer's disease and other cognitive disorders unit. Neurology department, Hospital Clínic, IDIBAPS, Barcelona, Spain

47 Neurological Tissue Bank of the Biobanc - Hospital Clinic-Institut d'Investigacions Biomediques August Pi i Sunyer (IDIBAPS), Barcelona, Spain

48 Department of Neurology, Medical University Sofia, Sofia, Bulgaria

49 Department of Cognitive Science and Psychology, New Bulgarian University, Sofia, Bulgaria

50 Department of Biochemistry, Molecular Medicine Center, Medical University, Sofia, Sofia, Bulgaria

51 Department of Neurological and Movement Sciences, University of Verona, Verona, Italy

52 Cyclotron Research Centre, University of Liege and Memory Clinic, CHU Liege, Belgium

53 Laboratory for Cognitive Neurology, Department of Neurology, University of Leuven and University Hospitals Leuven Gasthuisberg, Leuven, Belgium

54 Department of Neurology and Alzheimer Research Center, University of Groningen and University Medical Center Groningen, Groningen, The Netherlands

*** BELNEU consortium:** Jonathan Baets (Department of Molecular Genetics, VIB, Antwerp, Belgium; Institute Born-Bunge, University of Antwerp, Antwerp, Belgium; Antwerp University Hospital, Edegem, Belgium); Dirk Nuytten (Hospital Network Antwerp Stuivenberg , Antwerp, Belgium); Jan De Bleecker (University Hospital Ghent, Ghent, Belgium); Jan Versijpt, Alex Michotte (University Hospital Brussels, Brussels, Belgium); Adrian Ivanoiu (Saint-Luc University Hospital, Brussels, Belgium); Olivier Deryck, Bruno Bergmans (AZ Sint-Jan Brugge, Bruges, Belgium); Christiana Willems (Jessa Hospital, Hasselt, Belgium)

**** EU EOD consortium:** Anna Paterlini (Proteomics Unit, IRCCS Istituto Centro San Giovanni di Dio Fatebenefratelli, Brescia, Italy); Elena Alonso (Neurogenetics Laboratory, Division of Neurosciences, Center for Applied Medical Research, Universidad de Navarra, Pamplona, Spain); Manuel Seijo-Martínez (Department of Neurology, Hospital do Salnés, Pontevedra, Spain); Ramon Rene, Jordi Gascon, Jaume Campdelacreu (Department of Neurology, Hospital de Bellvitge, Barcelona, Spain); Madalena Martins, Mafalda Matos, André Janeiro, Ana Verdelho (Faculty of Medicine and Institute of Molecular Medicine, University of Lisbon, Portugal); Irene Piaceri (Department of Neurosciences, Psychology, Drug Research and Child Health (NEUROFARBA) University of Florence, Florence, Italy); Jenny Björkström, Anne Kinhult Ståhlbom, Marie Fallström (Department of Geriatric Medicine, Genetics unit, Karolinska University Hospital, Stockholm, Sweden); Charlotte Forsell, Anna-Karin Lindström, Lena Lilius (Karolinska Institutet, Department of Neurobiology, Care Sciences and Society (NVS), KI-Alzheimer Disease Research Center, Stockholm, Sweden); Inger Nennesmo (Department of Clinical Pathology and Cytology, Karolinska University Hospital, Stockholm, Sweden); Laura Fratiglioni (Aging Research Center, Department of Neurobiology, Care Sciences and Society (NVS), Karolinska Institutet and Stockholm University, Stockholm, Sweden);Tamara Eisele (Department of Psychiatry and Psychotherapy, Technische Universität München, 81675 München, Germany); Thomas Wieland (Institute of Human Genetics, Technische Universität München, 81675 Munich, Germany); Ricard Rojas-García, Marc Suárez-Calvet (Department of Neurology, IIB Sant Pau, Hospital de la Santa Creu i Sant Pau, Universitat Autònoma de Barcelona, Barcelona, Spain; Center for Networker Biomedical Research in Neurodegenerative Diseases (CIBERNED), Madrid, Spain); João Massano (Department of Neurology Centro Hospitalar São João, Portugal; Department of Clinical Neuroscience and Mental Health, Faculty of Medicine University of Porto, Porto, Portugal); Maria Helena Ribeiro (Faculty of Medicine, University of Coimbra, Coimbra, Portugal); Catarina Oliveira (Center for Neuroscience and Cell Biology, University of Coimbra, Coimbra, Portugal); Jose L Molinuevo, Anna Antonell (Alzheimer's disease and other cogntive disorders unit. Neurology department, Hospital); Robert Rusina, Zdenek Rohan (Department of Pathology and Molecular Medicine, Thomayer Hospital, Prague, Czech Republic); Robert Rusina (Center of Clinical Neurosciences, Department of Neurology, First Medical Faculty, Charles University in Prague, Czech Republic); Tiziana Cavallaro (Department of Neuroscience, AOUI Verona, Verona, Italy)

^✝^Correspondences to Prof. Dr. Christine Van Broeckhoven Ph.D., D.Sc.

E-mail: [christine.vanbroeckhoven@molgen.vib-ua.be](mailto:christine.vanbroeckhoven@molgen.vib-ua.be)

Neurodegenerative Brain Diseases Group

VIB Department of Molecular Genetics

University of Antwerp – CDE

Universiteitsplein 1, B-2610 Antwerp, Belgium

Tel: +32 3 265 1101

Fax: +32 3 265 1112

**SUPPLEMENTARY APPENDIX**

**Supplementary table 1. Descriptive characteristics of the EU EOD patient and control cohorts**

|  | **Total n** | **Familial n (%)** | **Disease onset or age at inclusion ± SD (years)** |
| --- | --- | --- | --- |
| **FTLD** | **1808** | **636 (35.2%)** | **63 ± 9.9** |
| Belgium | 357 | 120 (33.6%) |  |
| Italy | 500 | 153 (30.6%) |  |
| Germany | 263 | 40 (15.2%) |  |
| Spain | 368 | 144 (32.9%) |  |
| Portugal | 231 | 144 (62.3) |  |
| Sweden | 65 | 31 (47.7%) |  |
| Czech Republic | 20 | 1 |  |
| Bulgaria | 2 | 2 |  |
| Austria | 2 | 1 |  |
| **ALS** | **395** | **59 (14.9%)** | **58 ± 13.7** |
| Belgium | 135 | 26 (19.3%) |  |
| Germany | 101 | 15 (14.9%) |  |
| Spain | 94 | 5 (5.3%) |  |
| Italy | 32 | 3 (9.4%) |  |
| Bulgaria | 24 | 5 (20.8%) |  |
| Czech Republic | 4 | 0 |  |
| Portugal | 2 | 2 |  |
| Sweden | 2 | 2 |  |
| Austria | 1 | 0 |  |
| **Controls** | **3899** | NA | **66 ± 12.4**^a^ |
| Belgium | 982 | NA |  |
| Italy | 355 | NA |  |
| Spain | 244 | NA |  |
| Portugal | 44 | NA |  |
| German WES controls | 2274 | NA |  |

NA, not applicable. ^a^From the German WES controls no information on age was available.

**Supplementary table 2. Descriptives of the *SQSTM1* mutations described in the present study**

| **On cDNA level** | **Exon** | **On protein level** | **Functional domain** | **dbSNP** | **Novel** |
| --- | --- | --- | --- | --- | --- |
| NM_003900.4:c.47C>T | Exon 1 | NP_003891.1:p.Ala16Val |  |  | Novel |
| NM_003900.4:c.50C>T | Exon 1 | NP_003891.1:p.Ala17Val |  | rs141502868 | Novel |
| NM_003900.4:c.98C>T | Exon 1 | NP_003891.1:p.Ala33Val | PB1 | rs200396166 |  |
| NM_003900.4:c.240C>G | Exon 2 | NP_003891.1:p.Asp80Glu | PB1 |  | Novel |
| NM_003900.4:c.268G>A | Exon 2 | NP_003891.1:p.Val90Met | PB1 | rs181263868 |  |
| NM_003900.4:c.308A>G | Exon 3 | NP_003891.1:p.Lys103Arg | PB1 |  | Novel |
| NM_003900.4:c.319C>T | Exon 3 | NP_003891.1:p.Arg107Trp |  |  | Novel |
| NM_003900.4:c.350C>T | Exon 3 | NP_003891.1:p.Ala117Val |  | rs147810437 |  |
| NM_003900.4:c.352C>T | Exon 3 | NP_003891.1:p.Pro118Ser |  | rs200152247 | Novel |
| NM_003900.4:c.385G>A | Exon 3 | NP_003891.1:p.Asp129Asn | ZZ |  | Novel |
| NM_003900.4:c.457G>A | Exon 3 | NP_003891.1:p.Val153Ile | ZZ | rs145056421 |  |
| NM_003900.4:c.634C>T | Exon 4 | NP_003891.1:p.Arg212Cys |  | rs201263163 | Novel |
| NM_003900.4:c.656G>T | Exon 4 | NP_003891.1:p.Gly219Val |  |  | Novel |
| NM_003900.4:c.676T>C | Exon 5 | NP_003891.1:p.Ser226Pro | TRAF6 |  | Novel |
| NM_003900.4:c.683C>T | Exon 5 | NP_003891.1:p.Pro228Leu | TRAF6 | rs151191977 |  |
| NM_003900.4:c.694C>A | Exon 5 | NP_003891.1:p.Pro232Thr | TRAF6 |  | Novel |
| NM_003900.4:c.712A>G | Exon 5 | NP_003891.1:p.Lys238Glu | TRAF6 | rs11548633 |  |
| NM_003900.4:c.772G>A | Exon 6 | NP_003891.1:p.Asp258Asn |  |  | Novel |
| NM_003900.4:c.822G>C | Exon 6 | NP_003891.1:p.Glu274Asp | PEST1 | rs55793208 |  |
| NM_003900.4:c.838_840delGAG | Exon 6 | NP_003891.1:p.Glu280del | PEST1 |  | Novel |
| NM_003900.4:c.961C>T | Exon 6 | NP_003891.1:p.Arg321Cys | LIR | rs140226523 |  |
| NM_003900.4:c.962G>A | Exon 6 | NP_003891.1:p.Arg321His | LIR |  | Novel |
| NM_003900.4:c.986A>G | Exon 7 | NP_003891.1:p.Asp329Gly | LIR | rs148294622 | Novel |
| NM_003900.4:c.1043C>T | Exon 7 | NP_003891.1:p.Pro348Leu | PEST2 |  |  |
| NM_003900.4:c.1160C>T | Exon 7 | NP_003891.1:p.Pro387Leu | UBA |  |  |
| NM_003900.4:c.1175C>T | Exon 8 | NP_003891.1:p.Pro392Leu | UBA | rs104893941 |  |
| NM_003900.4:c.1186_1187insT | Exon 8 | NP_003891.1:p.(Glu396*) | UBA |  |  |
| NM_003900.4:c.1288A>C | Exon 8 | NP_003891.1:p.Thr430Pro | UBA |  | Novel |
| NM_003900.4:c.1316C>T | Exon 8 | NP_003891.1:p.Pro439Leu | UBA | rs199854262 |  |

*SQSTM1* variants observed in patients and in patients and controls are listed. NP_003891.1:p.Glu274Asp had a MAF of 0.024 in control persons, all other rare variants had a MAF<0.01.

**Supplementary table 3*. In silico* prediction of effect of patient-specific *SQSTM1* mutations on protein function**

| **SQSTM1 mutation** | **Associated phenotype** | **PMUT** | | | **SIFT** | | **Provean** | | **SNPs&GO** | |
| --- | --- | --- | --- | --- | --- | --- | --- | --- | --- | --- |
|  |  | **NN Output** | **Reliability** | **Prediction** | **SIFT score** | **Prediction** | **score** | **Prediction** | **Effect** | RI |
| p.Ala16Val^1^ | FTLD-ALS | 0,3865 | 2 | Neutral | 0.129 | Tolerated | -1.928 | Neutral | Disease | 2 |
| p.Asp80Glu^1^ | FTLD | 0,0637 | 8 | Neutral | 0.018 | Damaging | -3,156 | Deleterious | Neutral | 1 |
| p.Val90Met | FTLD | 0,3447 | 3 | Neutral | 0,141 | Tolerated | -1,249 | Neutral | Disease | 1 |
| p.Arg107Trp^1^ | ALS | 0,9649 | 9 | Pathological | 0,001 | Damaging | -5,019 | Deleterious | Disease | 7 |
| p.Asp129Asn^1^ | ALS | 0,2091 | 5 | Neutral | 0,000 | Damaging | -4,627 | Deleterious | Disease | 9 |
| p.Arg212Cys^1^ | FTLD-ALS | 0,9087 | 8 | Pathological | 0,077 | Tolerated | -1,700 | Neutral | Disease | 6 |
| p.Gly219Val^1^ | FTLD | 0,8947 | 7 | Pathological | 0,282 | Tolerated | -1,472 | Neutral | Neutral | 7 |
| p.Ser226Pro^1^ | FTLD | 0,7175 | 4 | Pathological | 0,020 | Damaging | -2,323 | Neutral | Disease | 4 |
| p.Pro228Leu | FTLD | 0,6086 | 2 | Pathological | 0,018 | Damaging | -4,323 | Deleterious | Neutral | 1 |
| p.Pro232Thr^1^ | FTLD | 0,4991 | 0 | Neutral | 0,065 | Tolerated | -3,642 | Deleterious | Disease | 0 |
| p.Asp258Asn^1^ | ALS | 0,4687 | 0 | Neutral | 0,000 | Damaging | -3,746 | Deleterious | Disease | 3 |
| p.Glu280del^1^ | FTLD | NA | NA | NA | NA | NA | -1,052 | Neutral | NA | NA |
| p.Arg321His | FTLD | 0,6996 | 3 | Pathological | 0,115 | Tolerated | -0,826 | Neutral | Neutral | 5 |
| p.Asp329Gly^1^ | FTLD | 0,5678 | 1 | Pathological | 0,540 | Tolerated | -0,626 | Neutral | Disease | 1 |
| p.Pro348Leu | FTLD | 0,8328 | 6 | Pathological | 0,025 | Damaging | -6,275 | Deleterious | Disease | 4 |
| p.Pro387Leu | FTLD | 0,9392 | 9 | Pathological | 0,028 | Damaging | -6,866 | Deleterious | Disease | 3 |
| p.(Glu396*) | FTLD | NA | NA | NA | NA | NA | NA | NA | NA | NA |
| p.Thr430Pro^1^ | FTLD | 0,7529 | 5 | Pathological | 0,047 | Damaging | -2,129 | Neutral | Disease | 6 |

^1^Indicates variants not previously associated with ALS, FTLD or PDB [8, 12, 18, 29, 30, 34].
